# Why should we continue to learn?

**Published:** 2017-05-12

**Authors:** Karl Golnik

**Affiliations:** 1 International Council of Ophthalmology Director for Education. Professor & Chairman, Department of Ophthalmology, University of Cincinnati & the Cincinnati Eye Institute, Ohio, USA.

**The World is changing and medical practice with it. Society expects healthcare workers not only to have knowledge and skills but to be competent, professional and up to date through life-long learning.**

**Figure:**
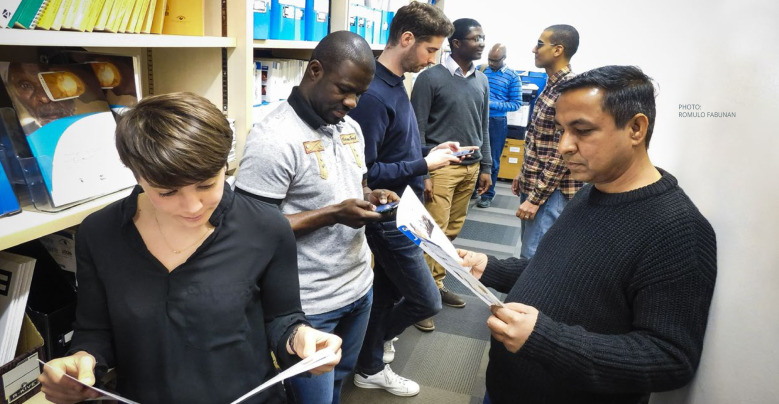
MSc Students 2017 – using multiple sources of information. UNITED KINGDOM

Formal under-graduate and post-graduate education prepares us with the basic “potential” to implement health care. Actual medical practice and responsibilities place demands on the individual to maintain a relevant level of knowledge, skills, attitudes and even behaviour, to improve standards of practice and patient care. The tensions that arise at a personal level are knowing what to learn, where to find it and how to record and measure it against peer groups or local standards.

## CME and CPD

Continuing Medical Education (CME) and Continuing Professional Development (CPD) are traditionally thought of as the methods by which medical providers maintain knowledge and skills that are up to date with the latest medical advances. In the past, CME events mostly consisted of meetings and printed material. It was not particularly individualised to specific needs and there were no requirements as to how much or what type of content was needed. There were also few rules regarding industry's (e.g. equipment and consumable manufacturers) participation in CME events.

Recently, increased emphasis has been placed on additional attributes thought essential to the provision of good health care, including professionalism and communication skills. In addition, societal expectations, proliferation of new technology and differences in individuals' needs have challenged the concept of CME. These factors have led to a new term “Continuing Professional Development” (CPD). CPD encompasses all of CME plus other aspects of being an effective medical care provider such as their professionalism, ethics and communication skills.

## Effective CPD

Effective CPD is a complex and multi-dimensional concept that directs a person to improve their practice, clinically and professionally within the dynamics of the local setting.

Therefore, CPD should be systematic, with formal programmes developed for all members of the health care teams. It should be comprehensive and include all competencies of the medical profession (medical expertise, collaboration, communication, leadership, advocacy, scholarship and professionalism). Ideally CPD programmes should be accredited and regulated by an outside review body to assure quality and unbiased education. Indeed, many countries now have organisations that assess and accredit CPD to assure high quality. In addition, effective CPD should:

- be individually relevant, addressing learning gaps
- produce change in the participant's practice
- have no commercial bias
- be required.

All of these concepts and advice on how to produce good CPD are described in detail in the International Council of Ophthalmology (ICO) Guide to Effective CME-CPD E-book (available as a free download at **www.icoph.org/downloads/ICOGuidetoEffectiveCPDCME.pdf**).

## CPD is essential for several reasons

- New treatments and concepts abound. Without CPD the eye care worker is woefully unprepared to practise appropriately.
- The world is continually shrinking as mobility of patients and physicians expands. Health care workers may be caring for patients from regions with unfamiliar diseases and diseases previously confined to a geographic region are now spreading to other parts of the world.
- Electronic medical records are making patient outcomes more transparent. This will drive health care workers to self-improvement through effective CPD.

**Figure:**
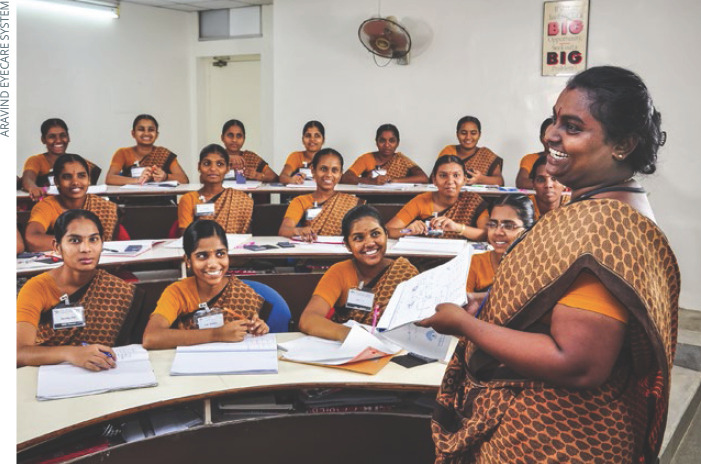
Nurses undergoing CPD together. Aravind Eye Care System. INDIA

All of these factors are increasing the demand for CPD. This demand produces revenue-generating opportunity for CPD providers (e.g. ophthalmic societies) to fund their non-CPD activities and achieve their goals.

**Why CPD matters**

CPD should matter to everyone involved in health care, including practitioners, managers, leaders of professional bodies, and members of ministries of health. New knowledge and required skills are exponentially increasing. On a personal note, the majority of ophthalmic procedures done today were not taught when I was in training and CPD is the mechanism by which I learn how to perform these skills.

## Universal health coverage and Sustainable Development Goals

Universal health coverage through increased availability, accessibility, quality of care and service provision can only be achieved through appropriately trained and equipped health personnel. In 2015 the United Nations General Assembly adopted 17 Sustainable Development Goals with the intent to end poverty, protect the planet and ensure prosperity for all. One of the challenges to achieving this is the lack of accessible health care around the world.

This is certainly true for eye health. Most countries don't have a sufficient number of ophthalmologists, optometrists and allied ophthalmic personnel.

Compounding the problem, skilled eye health care workers are often concentrated in large cities, leaving much of the country with poor access to eye care. In addition, skilled eye care workers may be unable to provide services within their scope of practice. For instance, although ophthalmologists provide surgical eye care, in some countries as few as 15% perform surgery.

Solutions to eye care access include the creation of more eye care workers, improved efficiency through eye care team training and expanding/improving abilities of current eye care workers. Quality of clinical and surgical care within a health system is dependent on practitioners engaging with established standards of practice, implementing governance and managing budgets, as part of their routine range of activities.

All of these solutions and activities require effective CPD.

## Conclusion

Thus, CPD is evolving and is crucial for the eye care worker's continuing competence. During training the principle of life-long learning must be emphasised. CPD allows staff to be confident in their skills and facilitates the efficiency, effectiveness and quality of the eye care team. It helps assure the best outcomes for the maximum number of patients and improve access to high quality eye care globally. The availability of effective CPD is improving but the demand and its increasingly mandatory nature will necessitate ever-expanding opportunities for quality CPD.

